# Single-session visuospatial task procedure to prevent childbirth-related posttraumatic stress disorder: a multicentre double-blind randomised controlled trial

**DOI:** 10.1038/s41380-023-02275-w

**Published:** 2023-09-27

**Authors:** Camille Deforges, Vania Sandoz, Yvonnick Noël, Valérie Avignon, David Desseauve, Julie Bourdin, Yvan Vial, Susan Ayers, Emily A. Holmes, Manuella Epiney, Antje Horsch

**Affiliations:** 1 Institute of Higher Education and Research in Healthcare, University of Lausanne, Lausanne, Vaud Switzerland; 2 Department Woman-Mother-Child, Lausanne University Hospital, Lausanne, Vaud Switzerland; 3 Department of Psychology, Rennes 2 University, Rennes, France; 4 Centre for Maternal and Child Health Research, City, University of London, London, UK; 5 Department of Psychology, Uppsala University, Uppsala, Sweden; 6 Department of Woman, Child and Teenager, Geneva University Hospitals, Geneva, Switzerland

**Keywords:** Psychiatric disorders, Psychology

## Abstract

Preventive evidence-based interventions for childbirth-related posttraumatic stress disorder (CB-PTSD) are lacking. Yet, 18.5% of women develop CB-PTSD symptoms following an unplanned caesarean section (UCS). This two-arm, multicentre, double-blind superiority trial tested the efficacy of an early single-session intervention including a visuospatial task on the prevention of maternal CB-PTSD symptoms. The intervention was delivered by trained maternity clinicians. Shortly after UCS, women were included if they gave birth to a live baby, provided consent, and perceived their childbirth as traumatic. Participants were randomly assigned to the intervention or attention-placebo group (allocation ratio 1:1). Assessments were done at birth, six weeks, and six months postpartum. Group differences in maternal CB-PTSD symptoms at six weeks (primary outcomes) and six months postpartum (secondary outcomes) were assessed with the self-report PTSD Checklist for DSM-5 (PCL-5) and by blinded research assessors with the Clinician-administered PTSD scale for DSM-5 (CAPS-5). Analysis was by intention-to-treat. The trial was prospectively registered (ClinicalTrials.gov, NCT03576586). Of the 2068 women assessed for eligibility, 166 were eligible and 146 were randomly assigned to the intervention (*n* = 74) or attention-placebo control group (*n* = 72). For the PCL-5, at six weeks, a marginally significant intervention effect was found on the total PCL-5 PTSD symptom count (β = −0.43, S.E. = 0.23, z = −1.88, *p* < 0.06), and on the intrusions (β = −0.73, S.E. = 0.38, z = −1.94, *p* < 0.0525) and arousal (β = −0.55, S.E. = 0.29, z = −1.92, *p* < 0.0552) clusters. At six months, a significant intervention effect on the total PCL-5 PTSD symptom count (β = −0.65, S.E. = 0.32, z = −2.04, *p* = 0.041, 95%CI[−1.27, −0.03]), on alterations in cognition and mood (β = −0.85, S.E. = 0.27, z = −3.15, *p* = 0.0016) and arousal (β = −0.56, S.E. = 0.26, z = −2.19, *p* < 0.0289, 95%CI[−1.07, −0.06]) clusters appeared. No group differences on the CAPS-5 emerged. Results provide evidence that this brief, single-session intervention carried out by trained clinicians can prevent the development of CB-PTSD symptoms up to six months postpartum.

## Introduction

Posttraumatic stress disorder (PTSD) has a worldwide lifetime prevalence of 3.9% [1] and is characterised by four symptom clusters, present at least one month after the triggering traumatic experience: intrusions, avoidance of trauma-related reminders, alterations in arousal, and negative cognitions and mood [2]. Childbirth-related PTSD (CB-PTSD) can, inter alia, occur after an unplanned caesarean section (UCS), following which 18.5% of women meet the diagnostic criteria for CB-PTSD [3], and an even larger proportion of them develops at least *some* CB-PTSD symptoms [4, 5]. Indeed, because it involves exposure to actual or imminent death and/or serious injury to the mother or infant, an UCS is a traumatic event as defined by the DSM-5 [2, 6]. Given that UCS are relatively frequent, it is crucial to improve care for the mothers concerned [7]. Indeed, CB-PTSD symptoms can negatively affect the entire family, including breastfeeding [8], child socio-emotional development [9, 10], future pregnancies [11, 12], and marital satisfaction [13]. Therefore, preventing CB-PTSD could also benefit children and co-parents. However, to date, evidence-based preventive interventions are lacking [14–16].

To develop such interventions, a relevant strategy is to specifically target the development of childbirth-related intrusive memories (IMs) [17]. IMs are one of the key symptoms of PTSD and CB-PTSD [17] They consist of repeated, involuntary, and distressing sensory-perceptual fragments of the trauma memory [17], which are hypothesised to predominantly result from excessive peritraumatic sensory (in particular visual) processing [18]. IMs can contribute to the development and maintenance of other PTSD symptoms, in addition to being distressing in their own right [17]. Therefore, preventing the development of IMs has the potential to prevent PTSD, including CB-PTSD [19].

Although the involved mechanisms are still debated, evidence suggests that a brief behavioural intervention procedure including a trauma reminder cue, mental rotation, and a visuospatial task, can successfully prevent IM development [20–22]. Indeed, engaging in a visuospatial task during the first few hours following a traumatic event, i.e., when memory is still malleable [23, 24], may take up the visuospatial information processing capacities necessary for memory’s consolidation of traumatic images, and thus prevent the subsequent development of IMs [20–22, 25].

Based on laboratory results [20, 21], two randomised controlled trials (RCTs) tested an intervention including a visuospatial task (i.e., trauma reminder cue, mental rotation, and then the computer game Tetris for 15 min) within six hours following trauma exposure in patients admitted to hospital emergency departments [26, 27]. Both RCTs reported that those who received the intervention had fewer IMs over the following week. However, the benefits of the intervention on broader PTSD symptoms were unclear.

In parallel, the intervention was tested by Horsch et al. [19] in a proof-of-principle RCT among 56 women after an UCS. The intervention group reported significantly fewer IMs over the first postpartum week than the treatment-as-usual control group. In per-protocol analyses, the intervention group also had less severe intrusion symptoms at one week postpartum and were less likely to have a CB-PTSD diagnosis at one month postpartum. Additionally, in these three RCTs, results suggested that the intervention may affect differently the PTSD symptom clusters [19, 26, 27]. A better understanding of the effect of the intervention on the different symptom clusters would thus be primordial in the prevention of CB-PTSD. Importantly, although offered after the traumatic birth occurred, this intervention is preventive given that women received it before developing CB-PTSD symptoms [28], which can only be assessed from one-month post-partum.

Despite their encouraging results, the above-mentioned studies have various major limitations. Participants and researchers were not blinded in any of these three RCTs, and the trials were powered to detect the effects of the intervention on the IMs but not on other PTSD symptoms [19, 26, 27]. Another point limiting the future clinical impact of these studies within hospital settings is that the intervention was delivered by researchers and/or mental health professionals whereas, in reality, patients are mainly seen by frontline healthcare workers. Furthermore, none of these studies followed their participants beyond five weeks post-trauma and, in Horsch et al. [19], the treatment-as-usual control group was passive. Taken together, the weaknesses of these three previous-mentioned studies considerably limit their clinical implications. At this stage, a double-blind RCT with an active control group, a longer follow-up, a sample size adapted to study the effects of the intervention on PTSD symptoms, and a procedure that directly includes frontline healthcare workers is therefore essential to establish the clinical benefits of the intervention.

The aim of the present RCT was to test the efficacy of a single-session behavioural intervention including a visuospatial task procedure on the secondary prevention [28] of maternal CB-PTSD symptoms (presence, severity) at six weeks (primary outcomes) and six months postpartum, as well as on the number of IMs over the first week following an UCS.

## Materials and methods

### Study design

The Swiss TrAumatic biRth Trial (START) was a two-arm (intervention *vs a*ttention-placebo), parallel-group, multicentre, double-blind, explanatory superiority RCT conducted in two Swiss University Hospitals. The study was preregistered on ClinicalTrials.gov before recruitment began (NCT03576586). The published study protocol was approved by the local ethics committee (2017–02142) [29]. A steering committee, including international perinatal mental health researchers and clinicians, advised on the conduct of the START trial from the study funding application to data analysis.

### Participants

All women aged ≥18 years giving birth were screened by trained clinicians (midwives and nurses) in the maternity units, for the following inclusion criteria: UCS at ≥34 weeks of gestation and gave birth to a live baby, as well as for the following exclusion criteria: insufficient French-speaking skills, established intellectual disability or psychotic illness, severe maternal or infant illness, infant required intensive care at birth [5], alcohol abuse and/or illegal drug use during pregnancy. Severe illnesses of the mother or infant included cancer, cardiovascular disease, severe neurodevelopmental difficulties, or malformations. This was assessed within the first 6 h by a member of the maternity team. Those women who provided informed consent (after having received oral and written information about the study) were then screened by a midwife or nurse as to whether they had perceived their childbirth as traumatic. Since all women had experienced an UCS, i.e., by definition a traumatic event, the addition of the fourth inclusion criterion was intended to filter the study population again and increase the likelihood of including women at high risk of developing CB-PTSD symptoms. Women were considered to have perceived their childbirth as traumatic when they scored ≥2 on a 7-point Likert scale (1=*not at all*, 7=*extremely*) separately for at least two out of four screening questions regarding perceived threat during childbirth: (1) Did you think that your life was in danger? (2) Did you think that your baby’s life was in danger? (3) Did you feel frightened during the birth? (4) Did you feel helpless during the birth? [29]. Only those who screened positively and maintained their agreement to participate in the study were then randomly allocated to one of the two study conditions. Recruitment occurred within the first six hours postpartum, on the recovery or postpartum ward of the hospital.

### Randomisation and masking

Participants were randomly assigned to a study condition (allocation ratio 1:1), using a computer-generated randomisation sequence, with blocks of 2, 4, and 6 over 144 participants per stratum (stratified by research centre). An independent statistician generated the sequence. The research team prepared beforehand and numbered consecutively sealed opaque envelopes by alternating between stratum. To ensure double-blinding, only the maternity clinical team (nurses or midwives) allocated the participants to a study condition by opening the envelope and delivering the *simple activity* to the participants. The term *simple activity* refers to *both* the visuospatial task and the attention-placebo control task and was always used when interacting with participants, who were, therefore, not informed about which of the allocated study conditions they received. The clinical team and participants were asked not to discuss with other members of the research team the type of *simple activity* performed. Thus, both the participants and the research team stayed blind to the group allocation until the last data collection point.

To avoid bias from the clinical team, maternity clinicians were trained through prior online training including detailed videos on all study procedures (screening, recruitment, simple activity, and completion of the first study measurements). This training systematically ended with a test of their understanding of the study procedures, followed by a contact from the research team to clarify any misunderstandings. Furthermore, clinicians were guided throughout the whole procedure with checklists and detailed instructions, including explanations to read to participants (standardised operating procedures).

A blind research assessor was individually assigned to each participant and was responsible for the follow-up and primary outcomes assessments. Due to indiscretions of the clinical team and/or participants (e.g., a midwife spontaneously telling the research assessor that the participant was now available as she had finished playing Tetris), the primary assessor’s masking was broken on seven occasions, following which another blind research evaluator was attributed to the participant. Moreover, one participant was not blind anymore at six weeks postpartum, and two at six months postpartum. The independent statistician in charge of the statistical analyses remained blind throughout the analyses.

### Procedure

The intervention and the attentional-control placebo tasks were both structured as simple cognitive tasks, lasting 15 min and delivered by the clinical maternity team whilst participants were still in their hospital bed at ≤6 h postpartum (T1). Participants allocated to the intervention group completed a cognitive visuospatial task (i.e., the computer game Tetris) on a handheld gaming device (Nintendo DS) [19]. A 3-min training preceded the intervention, allowing participants to familiarise themselves with the game and to actively use mental rotation while playing Tetris [19, 29]. Given the hospital setting, no additional memory reminder cue to the trauma was necessary, as the intervention took place in the same context where the trauma occurred [19]. Participants randomised to the attention-placebo control group were instructed not to sleep while keeping a written activity log (based on previous research [26]). They briefly reported the nature and duration of their activities (e.g., “*being with baby, 10* *min*”, “*lying on my bed, 5* *min*”, “*reading messages on my phone, 7* *min*”). Both activities were done once important routine care procedures had been completed. Additional information on the scientific rationale for selecting this attention-placebo control task is provided in the published study protocol [29].

Following the simple activity, participants completed a brief questionnaire regarding the intervention’s acceptability and their expectations of it. They then received instructions from a clinician on what IMs are and how to report them in a diary during the first week postpartum (T2) [19]. A blind member of the research team regularly checked the accurate diary completion with the participant during her maternity stay. At six weeks (T3) and six months (T4) postpartum, participants completed online self-report questionnaires and participated in a semi-structured diagnostic interview. At T4, financial compensation (75CHF) was given to participants for their time and effort. An independent, certified data monitoring body regularly checked that ethical and good research practices procedures were followed and confirmed the absence of adverse events related to the study.

### Outcomes

The primary outcomes consisted of differences in the presence and severity of maternal CB-PTSD symptoms between the intervention and the attention-placebo control groups at six weeks postpartum, assessed with the self-report PTSD Checklist for DSM-5 (PCL-5) [30] and the Clinician-administered PTSD scale for DSM-5 (CAPS-5) [31]. Instructions were updated so that the assessed symptoms were specifically related to the UCS. The PCL-5 is a 20-item self-report questionnaire measuring PTSD symptoms over the past month according to the DSM-5, on a 5-point Likert scale ranging from 0 to 4 (total range: 0–80; higher scores indicate greater symptom severity). It assesses the four symptom clusters of PTSD [30]. A symptom is present when the corresponding item is scored ⩾2, thus giving the possibility to compute both the total number of CB-PTSD symptoms and the number of symptoms per cluster. The internal validity of the PCL-5 was excellent (Cronbach’s α = 0.91 at T3 and 0.92 at T4). The CAPS-5 is a semi-structured diagnostic interview measuring PTSD symptoms [31]. It contains 20 items related to the four symptom clusters. Depending on symptom intensity and frequency, each symptom is scored from 0=*absent* to 4=*extreme/incapacity*. The presence of PTSD symptoms was assessed via the CAPS-5 total symptom count (i.e., the sum of items scoring ⩾ 2). The CAPS-5 showed good internal validity (Cronbach’s α = 0.88 at T3 and 0.86 at T4). Trained psychologists who were blind to which arm participants were in, conducted all the CAPS-5 interviews. Participants were reminded at the start of the CAPS-5 not to mention the *simple activity* they performed, in order to ensure the blinding of the CAPS-5 assessor. None of the participants revealed their group during these interviews.

Secondary outcomes included the differences in the presence and severity of CB-PTSD symptoms between the intervention and the attention-placebo control groups at six months postpartum, assessed in the same way as the primary outcomes. The number of IMs per day over the first postpartum week (T2) was assessed through the diary [19] with good convergent validity [32]. Due to an exceptional temporary lack of staff on the postpartum ward, the diary procedure was not followed correctly in one of the study centres. In agreement with the START steering committee and the secondary study centre, all diaries from participants (*n* = 30) at this study centre were excluded from the current paper. Since non-compliance with the diary procedure did not threaten the quality of the other study measurements, carried out at other time points by the research team, the rest of the data from this study centre were kept in the analyses that did not focus on IMs.

Other outcomes included a self-report questionnaire on the acceptability and expectancy of the intervention completed at T1. Sociodemographic and psychological information, such as maternal age, nationality, education, marital status, and prior psychological trauma were collected via a self-report questionnaire at T2. Obstetric data (gravidity, parity, blood loss >1 litre during childbirth) and infant data (Apgar score at 1 and 5 min, birth weight) were retrieved from hospital medical records. Based on previous clinical research on the same single-session behavioural intervention [19], no substantial risk was assumed to be associated with the intervention. Therefore, in agreement with the local ethics committees, no routine safety assessments took place.

### Choice of the primary measures

Both the PCL-5 and the CAPS-5 were chosen as primary outcome measures since they showed good psychometric properties [31, 33] and were previously used in CB-PTSD research [34]. Moreover, the CAPS-5 represents the gold-standard measure of PTSD diagnosis, while the PCL-5 measures PTSD symptoms.

### Statistical analysis

A sample size calculation, based on a previous proof-of-principle RCT [19], identified a sample size of *n* = 120 participants was necessary to have 80% power (α = 0.05) to detect a between-group difference of *d* = 0.30 on each of the primary outcomes, i.e., on the presence and severity of maternal CB-PTSD symptoms at six weeks postpartum. Predicting a 20% drop-out rate, we expected to recruit 144 women.

Parametric or non-parametric tests were chosen depending on whether the appropriate statistical assumptions were met. The sample was described with means (M) and standard deviations (SD), or median (Mdn) and interquartile ranges (IQR) when the data were not normally distributed according to Shapiro-Wilk tests. Group differences in the perception of the *simple activities* were examined with Mann-Whitney U tests. They were carried out on *n* = 136, corresponding to the number of participants who performed a simple activity and completed the acceptability-expectancy questionnaire, regardless of whether they completed other assessments.

PCL-5 and CAPS-5 symptom counts were taken as the main dependent measure in the follow-up analyses. Besides being clinically relevant, they have the advantage of including both disorder presence and its severity assessment. The distributions of symptom counts were compared between groups, first on the total symptom count and then within each DSM-5 cluster of symptoms. A well-known aspect of such data is the zero-inflation phenomenon: a mass of zero counts is often apparent, exceeding what would be expected from standard count models, resulting in a bimodal distribution. Ignoring this structural aspect of data may lead to bias in regression coefficient estimation [35]. Another inherent aspect of such counts is that, as within-subject counts, they are dependent, and under- or overdispersion is to be expected. Both issues were addressed in the analyses by using zero-inflated count models, with various degrees of flexibility to model under- or overdispersion: The Poisson, Negative Binomial I (linear parameterisation), Negative Binomial II (quadratic parameterisation), and Generalised Poisson [36] distributions were systematically compared for each count variable, using the R software package version 4.2.1 [37], and the glmmTMB R package [38]. A log link was used in all models.

A model comparison approach was adopted, where all three components (distribution choice, variable inclusion in the conditional model, and variable inclusion in the zero-inflation part) were systematically varied. All possible sub-models, including or not the group variable in the conditional model, including or not a zero-inflation component were fitted. When a variable was included in the conditional part of the model, it was also included in the zero-inflation component. The log-sample size was used as an offset in all models, to account for unequal sample sizes. For each dependent variable, the best model was selected using the Akaike Information Criterion (AIC) [39]. which showed good power in the detection of zero-inflation and under- overdispersion in simulation studies [35]. The intervention effect was assessed through examination of the group variable coefficient within the model if present. Note that this amounts to retaining an effect as significant if it both (i) was retained in the model selection phase and (ii) displayed a significant coefficient within this model. The numbers of eligible participants for these analyses were *n* = 128 at T3, and *n* = 113 at T4. Given there were missing responses for six participants at T3 and five at T4 on at least one PCL-5 sub-score, the analyses included *n* = 122 participants at T3 and *n* = 108 participants at T4. Concerning the CAPS-5, the exclusion of participants with missing responses resulted in a total of *n* = 116 participants (*n* = 54 control, *n* = 62 intervention) at T3, and *n* = 100 (*n* = 49 control, *n* = 51 intervention) at T4.

A similar approach was adopted for analysing the diary data (*n* = 96), consisting of daily counts of trauma-related IMs, during the first week postpartum. All four candidate distributions were fitted within a generalised linear mixed model regression, with time (i.e., days 1–7) and the group as the main explanatory variable, and subjects as a random intercept variable.

## Results

**Fig. 1:**
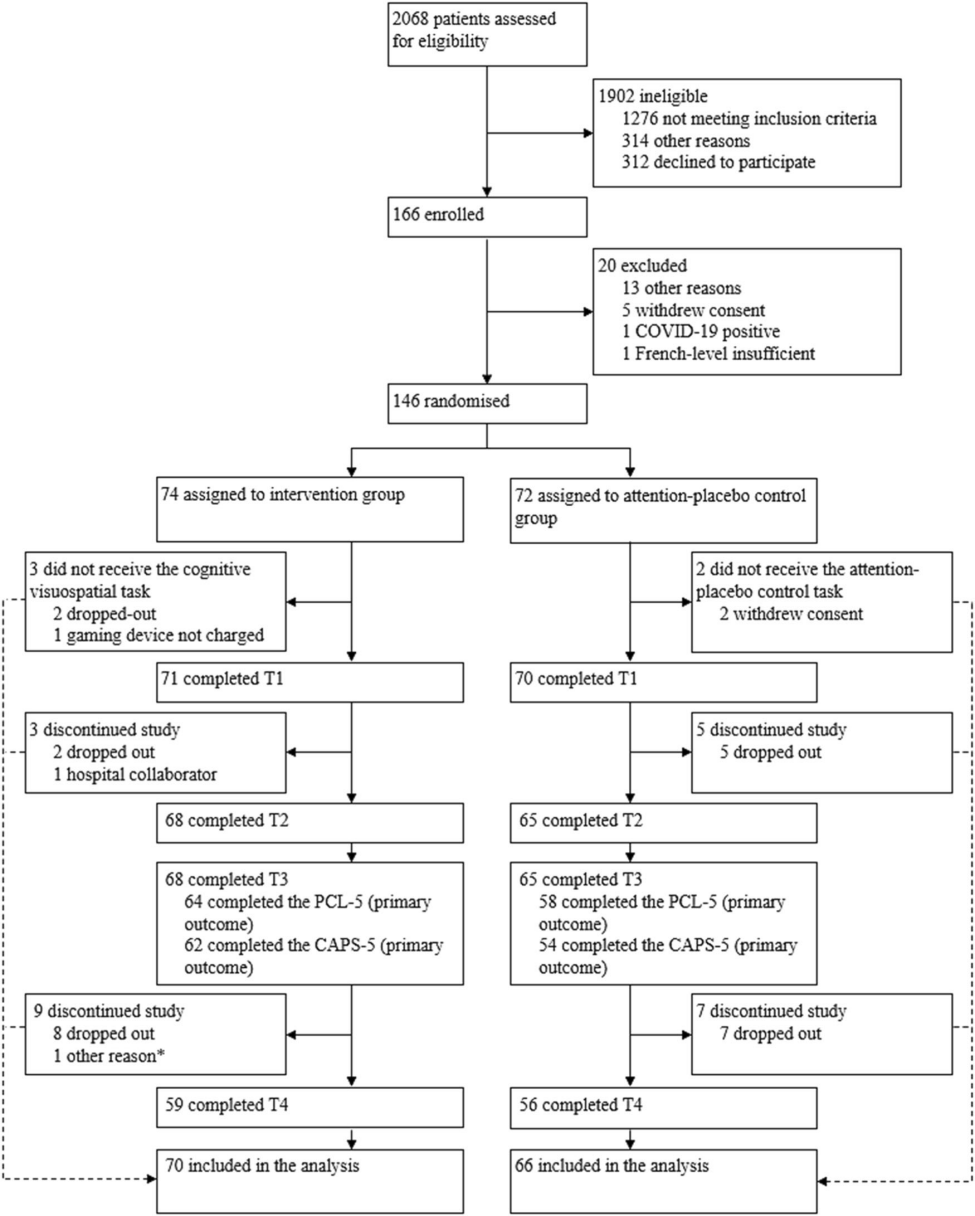
Trial profile.

Recruitment occurred between August 2nd 2018 and Oct 10th 2021 in the main study centre, and between March 3rd 2019 to July 29th 2020 in the secondary study centre. In both centres, recruitment was paused between March 16th 2020 and June 8th 2020 due to restrictions related to the COVID-19 pandemic. Final follow-up data were collected on Feb 8th 2022 for the main study centre and on Feb 7th 2021 for the secondary study centre. A total of 2068 women gave birth in the two study centres over the study period: 1902 of them were finally ineligible and 166 were enrolled in START, with 74 allocated to the intervention group, 72 to the attention-placebo control group, and 20 excluded between the study enrolment and randomisation for various reasons (Fig. 1). Among the intervention group, three participants did not receive the intervention, three dropped out of the study between T1 and T2, and eight between T3 and T4. Regarding the control group, two participants did not complete the attention-placebo control task, while five dropped out between T1 and T2 and seven between T3 and T4. In the intervention group, 64 and 62 participants, completed the PCL-5 and the CAPS-5 at T3, respectively, and 58 and 52 in the control group. The trial ended when a sufficient sample size for the primary outcomes was reached. In total, 136 participants were included in the analyses. Sociodemographic and clinical properties of the sample are displayed in Table 1.

**Table 1:** Sociodemographic and clinical characteristics of participants who completed the childbirth-related posttraumatic stress disorder assessments or the intrusive memory diaries (*n* = 133).

Sample characteristics	Frequency (n, %)	Median (IQR) or Mean (SD)^a^
*Sociodemographic characteristics*		
Age at the time of childbirth (years)		33.9 (4.2)^△^
Nationality^b^		
Swiss	55 (41.4)	
Other European	36 (27.1)	
Non-European	7 (5.3)	
Education^b^		
Primary school	1 (0.8)	
Secondary school or equivalent	9 (6.8)	
Apprenticeship	33 (24.8)	
University	69 (51.9)	
Marital status at one week postpartum^b^		
In a relationship	74 (55.6)	
Single or separated	38 (28.6)	
*Obstetrical variables*		
Gravidity		
First pregnancy	59 (44.4)	
Second pregnancy	45 (33.8)	
Third pregnancy	19 (14.3)	
Fourth pregnancy or more	10 (7.5)	
Parity		
Nulliparous	67 (50.4)	
Parous	66 (49.6)	
Gestational age (weeks)		40 (2.0)
Pregnancy type		
Single	131 (98.5)	
Multiple	2 (1.5)	
Blood loss > 1 litre during childbirth^b^		
Yes	10 (7.5)	
No	112 (84.2)	
*Neonatal variables*^c^		
Apgar score^d^		
Apgar score 1 min		9 (2)
Apgar score 5 min		10 (1)
Birth weight (grams)		3273 (499.4)^△^
*Mental health variables*		
Prior psychological trauma^b,e^		
Yes	56 (42.1)	
No	56 (42.1)	
Depression symptoms at six weeks postpartum (EPDS score)		5 (7)
Probable depression^f^	19 (14.3)	
Anxiety symptoms at six weeks postpartum (HADS-A score)		5 (4)
Screening questions^g^		
Question 1 (life in danger)		2 (2)
Question 2 (baby’s life in danger)		3 (4)
Question 3 (frightened)		3 (4)
Question 4 (helpless)		5 (4)

*EPDS* Edinburgh Postnatal Depression Scale (range 0–30); *HADS-A* Hospital Anxiety and Depression Scale (anxiety subscale only, range 0–21).

^a^Median and interquartile ranges are reported if the data did not follow a normal distribution according to a Shapiro–Wilk test and means and standard deviations (indicated with a^△^) otherwise.

^b^Total of % does not equal 100 because of missing values (*n* = 98 for nationality, *n* = 112 for education, marital status, and prior psychological trauma, *n* = 122 for blood loss, *n* = 119 for EPDS).

^c^In case of multiple pregnancies, data of the firstborn child was used.

^d^Apgar score ranges from 0 to 10, with a higher score referring to higher infant extra-uterine functioning.

^e^Assessed at six weeks postpartum with a dichotomous self-report question: “In the past, have you experienced any traumatic event(s) (e.g., death of a relative, car accident, physical or sexual assault, etc)?”.

^f^EPDS score > 10.5.

^g^The four screening questions are detailed in the Methods.

Participants’ expectations regarding the impact of the *simple activity* on the number of their IMs were similar, regardless of their group (*p* = 0.3225) (Table 2). Groups did not differ in their evaluation of the activity’s characteristics or their willingness to perform the simple activity again in the future (all *p* > 0.05, Table 2).

**Table 2:** Expectancies about the intervention: participant’s perception of the *simple activity*, in each group, regardless of their completion of other study outcomes (*n* = 136, of which *n* = 58 in the control group and *n* = 64 in the intervention group).

	Cognitive visuospatial task^a^ Intervention group	Written activity log^a^ Control group		
	Mdn (IQR)	Mdn (IQR)	*U*	*p*
Expected changes in childbirth-related IMs due to the activity^a^	10 (2)	10 (3)	2130	0.3225
Easiness of the activity^b^	7 (4)	7 (4)	2374	0.9843
Helpfulness of the activity^b^	4 (5)	5 (4)	2223.5	0.5019
Burdensomeness of the activity^b^	2.5 (4)	2.5 (4)	2366	0.9562
Willingness to do the activity after a future UCS^c^	8 (3)	7 (4)	2156.5	0.3986

^a^To what extent participants expect the *simple activity* to increase or decrease their number of childbirth-related intrusive memories (0 = *Extreme decrease*; 20 = *Extreme increase*, 0 corresponding to *No effect*).

^b^To what extent participants find the activity easy, helpful, burdensome (1 = Not at all easy/helpful/burdensome; 10 = Extremely easy/helpful/burdensome).

^c^To what extent would participants be willing to do this activity again, if they had another UCS and the activity was scientifically shown to be useful (1 = Not at all; 10 = Absolutely).

^d^To what extent would participants recommend the activity to a friend who has had an UCS (1 = Not at all; 10 = Absolutely).

Both groups were equally well represented within centres (χ²(1) = 0.22, *p* < 0.6388), and PCL-5 total PTSD symptom counts were homogeneously distributed across centres (χ²(13) = 7.88, *p* < 0.8514).

Zero-inflated generalised linear models for count data were fitted to the PCL-5 data. Count variables, selected distribution models, and effects are summarised in Table 3, for T3 and T4. Detailed tables of coefficients for all best models are provided in the Supplementary Information, section I.

**Table 3:** Intervention effects on PTSD symptoms reported in the PTSD Checklist for DSM-5 at six weeks (*n* = 122, of which *n* = 58 in the control group and *n* = 64 in the intervention group) and six months postpartum (*n* = 108, of which *n* = 50 in the control group and *n* = 58 in the intervention group).

	Symptom counts	Distribution	Zero inflation	Intervention effect
Six weeks postpartum (T3)	Total	Negative Binomial (I)	None	*β* = −0.43, S.E. = 0.23, *z* = −1.88, *p* < 0.06
	Intrusions	Negative Binomial (I)	None	*β* = −0.73, S.E. = 0.38, *z* = −1.94, *p* < 0.0525
	Avoidance	Generalised Poisson	Yes	None
	Negative alterations in cognition and mood	Poisson	Yes	None
	Arousal	Negative Binomial (II)	None	*β* = −0.55, S.E. = 0.29, *z* = −1.92, *p* < 0.0552
Six months postpartum (T4)	Total	Negative Binomial (II)	None	*β* = −0.65, S.E. = 0.32, *z* = −2.04, *p* = 0.041, 95% CI [−1.27, −0.03]
	Intrusions	Poisson	Yes	*β* = −0.72, S.E. = 0.41, *z* = −1.77, *p* = 0.0771
	Avoidance	Generalised Poisson	Yes	None
	Negative alterations in cognition and mood	Poisson	Yes	*β* = −0.85, S.E. = 0.27, *z* = −3.15, *p* = 0.0016, 95% CI [−1.38, −0.32]
	Arousal	Poisson	Yes	*β* = −0.56, S.E. = 0.26, *z* = −2.19, *p* < 0.0289, 95% CI [−1.07, −0.06]

A negative coefficient corresponds to a lowered severity in the intervention group. No Group effect was retained in the zero-inflation part of these models, and zero inflation, when detected, is constant across groups.

At T3, a marginally significant intervention effect was found on the total PCL-5 PTSD symptom count (*β* = −0.43, S.E. = 0.23, *z* = −1.88, *p* < 0.06). Detailed analysis of between-group differences on the four symptom clusters revealed marginally significant intervention effects on intrusions (*β* = −0.73, S.E. = 0.38, *z* = −1.94, *p* < 0.0525) and arousal (*β* = −0.55, S.E. = 0.29, *z* = −1.92, *p* < 0.0552), but no significant effects for avoidance and alterations in cognition and mood.

**Fig. 2:**
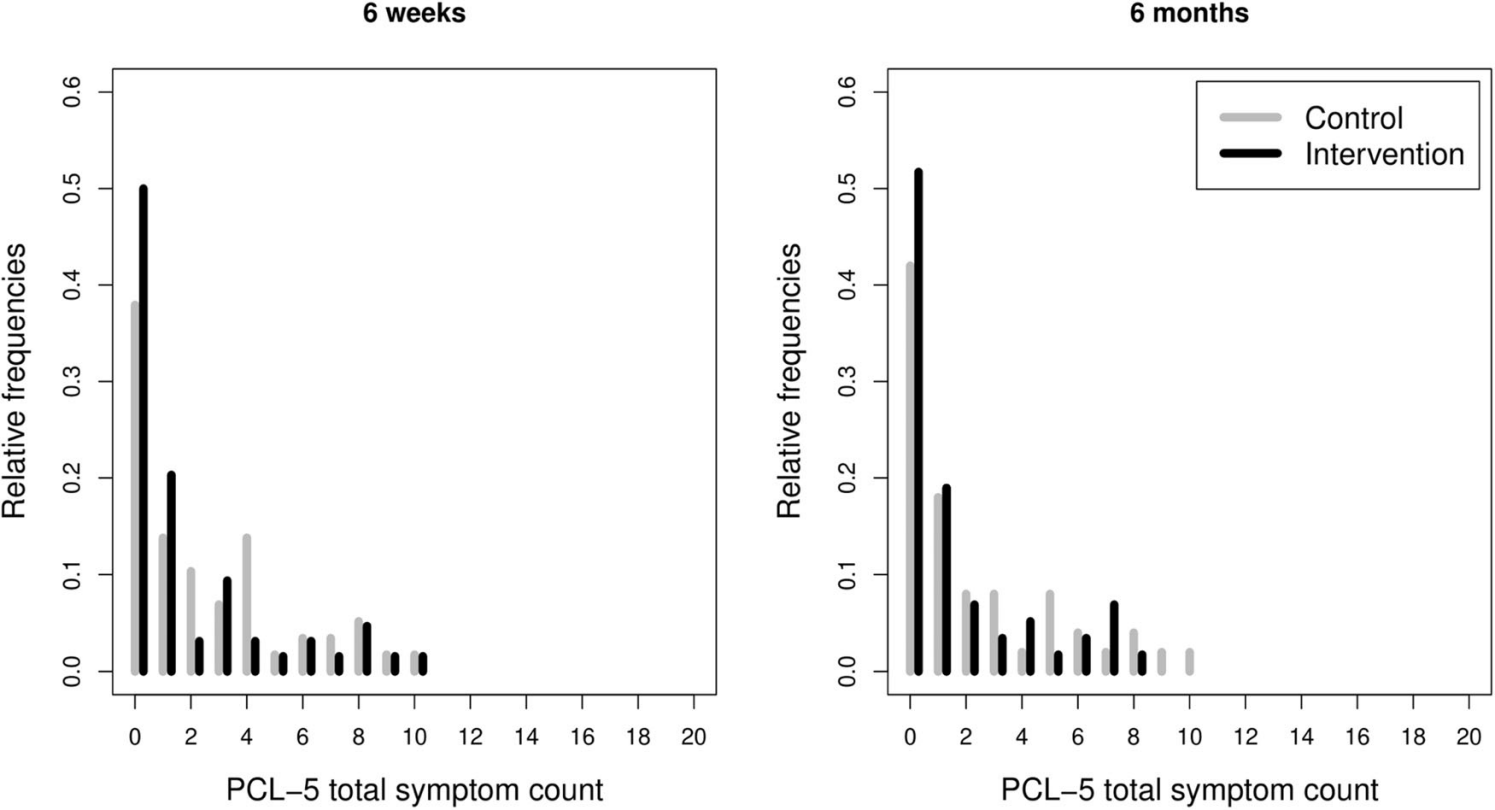
Distributions of the childbirth-related posttraumatic stress disorder symptom counts in the control and intervention groups at six weeks (*n* = 122) and six months (*n* = 108) postpartum, as measured by the self-report PTSD Checklist for DSM-5 (PCL-5).

At T4, the intervention had a significant and positive effect on the total PCL-5 PTSD symptom count (*β* = −0.65, S.E. = 0.32, *z* = −2.04, *p* = 0.041) i.e., with lower symptoms in the intervention group. A contraction towards zero of the symptom count distribution is noticeable in the intervention group at both T3 and T4 (Fig. 2). Within the four symptom clusters, significant effects on alterations in cognition and mood (*β* = −0.85, S.E. = 0.27, *z* = −3.15, *p* = 0.0016) and arousal (*β* = −0.56, S.E. = 0.26, *z* = −2.19, *p* < 0.0289) were found, but not on avoidance. A group effect, although retained in the final model for intrusions, did not prove significant at the coefficient level. Similar analyses performed on the CAPS-5 PTSD scores did not reveal any significant effects, neither on the total symptom count nor on the cluster counts.

As for the course of IMs, Generalised Poisson model with no zero-inflation fitted the diary data better than any other candidate model. Although the observed daily averages of IM counts appeared modest, a significant Group x Day interaction was found, suggesting that, within a globally decreasing frequency pattern, the intervention group displayed a lower initial level of intrusion (see Supplementary Information, section II).

Overall, note that no adverse events were reported by participants in this study.

## Discussion

This double-blind multicentre RCT with an active placebo control group tested the effect of a single-session behavioural intervention for IMs including a visuospatial task, delivered by trained maternity clinicians, to prevent the development of broader CB-PTSD symptoms. Participants who received the intervention tended to report fewer CB-PTSD symptoms at six weeks postpartum. This was not only the case for the total number of symptoms but also for intrusions and arousal symptoms although, importantly, *p* values were comprised between 0.05 and 0.06 for these three outcomes. Furthermore, participants reported significantly fewer symptoms of CB-PTSD (all clusters combined), including fewer symptoms of intrusions, altered cognitions and mood, and arousal, at six months postpartum. Note that these effects appeared even though participants in the intervention group had similar expectations concerning the intervention or placebo activity as those in the control group. However, no differences were found with the clinician-administered PTSD scale. Importantly, this was the first study testing such an intervention that followed up participants over a long period. Results suggest the intervention had a greater effect at six months than six weeks postpartum, whereas the benefits of psychological interventions usually tend to diminish over time. This pattern may possibly reflect the fact that PTSD symptoms are thought to contribute insidiously to the development and maintenance of each other [17, 40], thus an intervention preventing such symptoms at six weeks post-trauma would, through a snowball effect, have even greater long-term benefits. However, given this is the first time that such a long follow-up of the intervention effect was carried out, we need to remain cautious with its interpretation.

Given the rigorous design of this study, these results have clinical implications and are a further step towards an implementation study into routine clinical care. This intervention, carried out by first line clinicians, is brief and fully compatible with routine clinical care. Our results cautiously confirm its efficacy in the secondary prevention of CB-PTSD symptoms development, as already reported in a previous proof-of-principle RCT with a passive control group [19], while also suggesting that its benefits are particularly apparent at six months postpartum. Note that, in the present study, differences were only found for self-reported CB-PTSD symptoms, and not in the clinician-rated assessments. As self-reported PTSD symptoms tend to be more severe than clinician-reported symptoms [41], it is likely that a floor effect prevented group differences from being also observed in clinical assessments. Another hypothesis is that the participants minimised their symptoms to clinicians because of avoidance or shame of suffering from the birth of their child, while this event is socially considered to be a positive one. In any case, the absence of group differences in clinical interviews warrants caution in interpreting the effects of the intervention.

In line with our hypotheses, the intervention group reported half as many IMs as the control group during the first postpartum day. However, unexpectedly, the groups did not differ over the rest of the diary period. This contrasts with previous studies reporting that such visuospatial interventions led to fewer IMs within the week following the traumatic event [20, 27], including an UCS [19]. A striking difference with Horsch et al. [19] is that the total number of IMs reported by participants was considerably lower in the present study (i.e., M = 9.22, SD = 10.69 IMs per diary in the control group of Horsch et al. [19] whereas, in our study, the average daily number of IMs was ≤0.5 from day two to seven). These very low numbers of IMs may have led to a floor effect as well, preventing group differences from being observed. Participants may have reported fewer IMs for several reasons: first, in the proof-of-principle RCT [19], recruitment was done by psychologists and not first-line clinicians. Midwives and nurses may have been more protective, i.e., had less confidence in the intervention than mental health professionals and did not feel that it was worth adding research-related procedures to sensitive clinical care. Clinicians’ attitudes toward the intervention could typically be investigated in future implementation studies. On the contrary, psychologists’ confidence in the intervention may have induced a placebo effect contribution to the larger effect sizes in Horsch et al. [19]. A second hypothesis would be that IM diaries were not presented in the same way in both studies, as in our case this was done by trained maternity clinicians rather than psychologists [19], leading to potential under-reporting of IMs. Finally, involving maternity clinicians possibly made them more sensitive to trauma-related topics, as they all had been trained for this study, and this may have somehow improved their way of caring for women having UCS, thus resulting in fewer IMs in participants.

Compared to midwife-led debriefing, currently the main technique used to prevent CB-PTSD but whose efficacy remains unclear [42], the intervention proposed here does not involve narrating and reliving difficult memories, thus making it less distressing. Moreover, it does not require any specific language skills and could therefore benefit non-native speaking women. Additionally, the intervention can be offered by any trained healthcare worker, making it widely accessible and easy to integrate into routine care. For the same reason, this type of single-session intervention could be offered on a large scale and benefit other trauma-exposed populations, such as road accident victims [27] or police and firemen. However, future studies are needed to further investigate the cost-effectiveness of such interventions. Similarly, the benefits of the intervention on the whole family, particularly on the partner’s mental health, the couple’s relationship, and the child’s development, are currently being investigated [29].

This RCT had many strengths, including double-blinding, an active control group, and a prospective trial registration. The intervention was carried out by rigorously trained first-line clinicians. Furthermore, participants were followed-up until six months post-intervention. The analyses, conducted by a senior statistician, were based on innovative models and included covariates of major clinical and statistical importance. However, several limitations need to be highlighted. For example, although validated, the self-report questionnaires used may be subject to well-known biases, such as social desirability or recall bias. Furthermore, for ethical reasons, we did not include mothers who had given birth before 34 weeks of gestation or whose baby had died, and it was not necessarily possible to approach women with the most severe complications as they still underwent important care procedures, were too distressed, or experienced severe pain within the six first hours. This limits the generalisability of the results. As for the attention-placebo control task, unlike the intervention, it was not computer-based. However, in the absence of a validated task excluding any visuospatial processing, this comparator appeared relevant since it had already been used in a previous trial [26] and shared several core characteristics with the intervention (duration, involvement of frontline clinicians, structured instructions). Another limitation of the study is that, despite selecting women who not only experienced a traumatic event but also perceived a threat to themselves and/or their child during childbirth, the study sample did not appear to be highly distressed and therefore developed few IMs and symptoms of CB-PTSD. Moreover, the blinding of members of the research team was broken on seven occasions due to spontaneous indiscretions by the clinicians. As this always occurred in the first few days postpartum, the follow-up of the participant concerned was systematically entrusted to another, still blind, member of the research team, and this had therefore no impact on the validity of the assessments of CB-PTSD symptoms. Finally, the COVID-19 pandemic may have potentially affected study results, as the significant changes in maternity care including, for example, limited access to the labour and postpartum ward for partners, may have impacted the experience of the UCS.

Future research should investigate the implementation of a care pathway involving the routine screening of women with regard to perceived threat during childbirth and then systematically offering this single-session brief intervention to those who screened positively. This would also require investigation of how well the perception of threat to one’s own life and/or to that of one’s infant during childbirth (as applied in the current study) predicts the development of maternal CB-PTSS, which in turn could be the basis for the development of an evidence-based brief screening tool.

Overall, this double-blind multicentre RCT with an active placebo group provides evidence that a single-session intervention including a visuospatial task can prevent the development of CB-PTSD symptoms up to six months postpartum. Crucially, this intervention, carried out by trained clinicians, is brief, highly acceptable, and easy to integrate into routine clinical care. Future research may thus evaluate its implementation.

### Supplementary information

Supplementary information

## Data Availability

Not all participants agreed to share their data with persons outside of the START Research Consortium. The study protocol is available at 10.1136/bmjopen-2019-032469. The ethical study protocol, including the statistical analysis plan, statistical code, and data dictionary are available on request from the corresponding author.
