# Supplementary material for: Single-session visuospatial task procedure to prevent childbirth-related posttraumatic stress disorder: a multicentre double-blind randomised controlled trial
Source: Mol Psychiatry. 2023 Sep 27;28(9):3842–50. doi: 10.1038/s41380-023-02275-w (PMC10730415; doi:10.1038/s41380-023-02275-w)
Supplement: Supplementary file 1 — Supplementary information [file 41380_2023_2275_MOESM1_ESM.docx]

**Supplementary Information**

#

# Coefficients tables

Tables of coefficients for all models revealing an intervention effect are detailed below. Note that, because zero-inflated generalised linear models are two-tier models, both parts of which are not independent, a well-founded definition of a factor effect size in these models is not available, as far as we know. Confidence intervals on the coefficients are provided in the last columns of each table (Supplementary Tables 1 to 3).

**Supplementary Table 1. Negative binomial regression coefficients for the PTSD Checklist for DSM-5 (PCL-5) total PTSD symptom count at six months postpartum (dispersion parameter: 0.454).**

|  | **Estimate** | **Std. Error** | **z value** | **Pr(>\|z\|)** | **2.5%** | **97.5%** |
| --- | --- | --- | --- | --- | --- | --- |
| Conditional intercept | -2.97 | 0.23 | -13.05 | 0.0001 | -3.42 | -2.53 |
| Intervention group | -0.65 | 0.32 | -2.04 | 0.05 | -1.27 | -0.03 |

**Supplementary Table 2.** **Poisson regression coefficients for the PCL-5 arousal symptom count at six months postpartum.**

|  | **Estimate** | **Std. Error** | **z value** | **Pr(>\|z\|)** | **2.5%** | **97.5%** |
| --- | --- | --- | --- | --- | --- | --- |
| Conditional intercept | -3.17 | 0.17 | -18.76 | 0.0001 | -3.5 | -2.84 |
| Intervention group | -0.56 | 0.26 | -2.19 | 0.03 | -1.07 | -0.06 |
| ZI Intercept | 0.17 | 0.25 | 0.69 | 0.49 | -0.32 | 0.66 |

*Note*. ZI = Zero inflation.

**Supplementary Table 3**. **Poisson regression coefficients for the PCL-5 negative alteration in cognition and mood symptom count at six months postpartum.**

|  | **Estimate** | **Std. Error** | **z value** | **Pr(>\|z\|)** | **2.5 %** | **97.5 %** |
| --- | --- | --- | --- | --- | --- | --- |
| Conditional intercept | -2.98 | 0.16 | -18.58 | 0.0001 | -3.3 | -2.66 |
| Intervention group | -0.85 | 0.27 | -3.15 | 0.01 | -1.38 | -0.32 |
| ZI Intercept | 0.36 | 0.25 | 1.49 | 0.14 | -0.12 | 0.85 |

*Note*. ZI = Zero inflation.

# Analysis of the evolution of intrusive memories (IMs) within the first postpartum week

**Supplementary Table 4. Daily averages of intrusive memory counts (n = 96, of which n = 45 in the control group and n = 51 in the intervention group).**

| **Group / Day** | **1** | **2** | **3** | **4** | **5** | **6** | **7** |
| --- | --- | --- | --- | --- | --- | --- | --- |
| Control | 0.84 | 0.5 | 0.42 | 0.21 | 0.2 | 0.08 | 0.18 |
| Treatment | 0.44 | 0.45 | 0.47 | 0.36 | 0.31 | 0.18 | 0.2 |

**Supplementary Table 5**. **Analysis of deviance table for the IM data (n = 96).**

| **Model** | **Df.** | **AIC** | **BIC** | **Log likelihood** | **Deviance** | **Chisq.** | **Df.** | **Pr(>Chisq)** |
| --- | --- | --- | --- | --- | --- | --- | --- | --- |
| Constant | 3 | 857.35 | 870.65 | -425.68 | 851.35 |  |  |  |
| Day | 4 | 830.05 | 847.77 | -411.02 | 822.05 | 29.31 | 1 | 0.0001 |
| Day + Group | 5 | 831.89 | 854.04 | -410.94 | 821.89 | 0 | 1 | 0.7 |
| Day x Group | 6 | 827.7 | 854.27 | -407.38 | 815.7 | 6.2 | 1 | 0.02 |

**Supplementary Table 6.** **Regression coefficients for the Generalised Poisson model of the evolution of intruding memories counts, during the first postpartum week (n = 96).**

| **Model term** | **Estimate** | **Std. Error** | **z value** | **Pr(>\|z\|)** |
| --- | --- | --- | --- | --- |
| (Intercept) | -0.61 | 0.32 | -1.88 | 0.06 |
| Day | -0.34 | 0.07 | -5.19 | 0.0001 |
| TreatGroup | -0.79 | 0.31 | -1.82 | 0.07 |
| Day:TreatGroup | 0.21 | 0.09 | 2.47 | 0.02 |


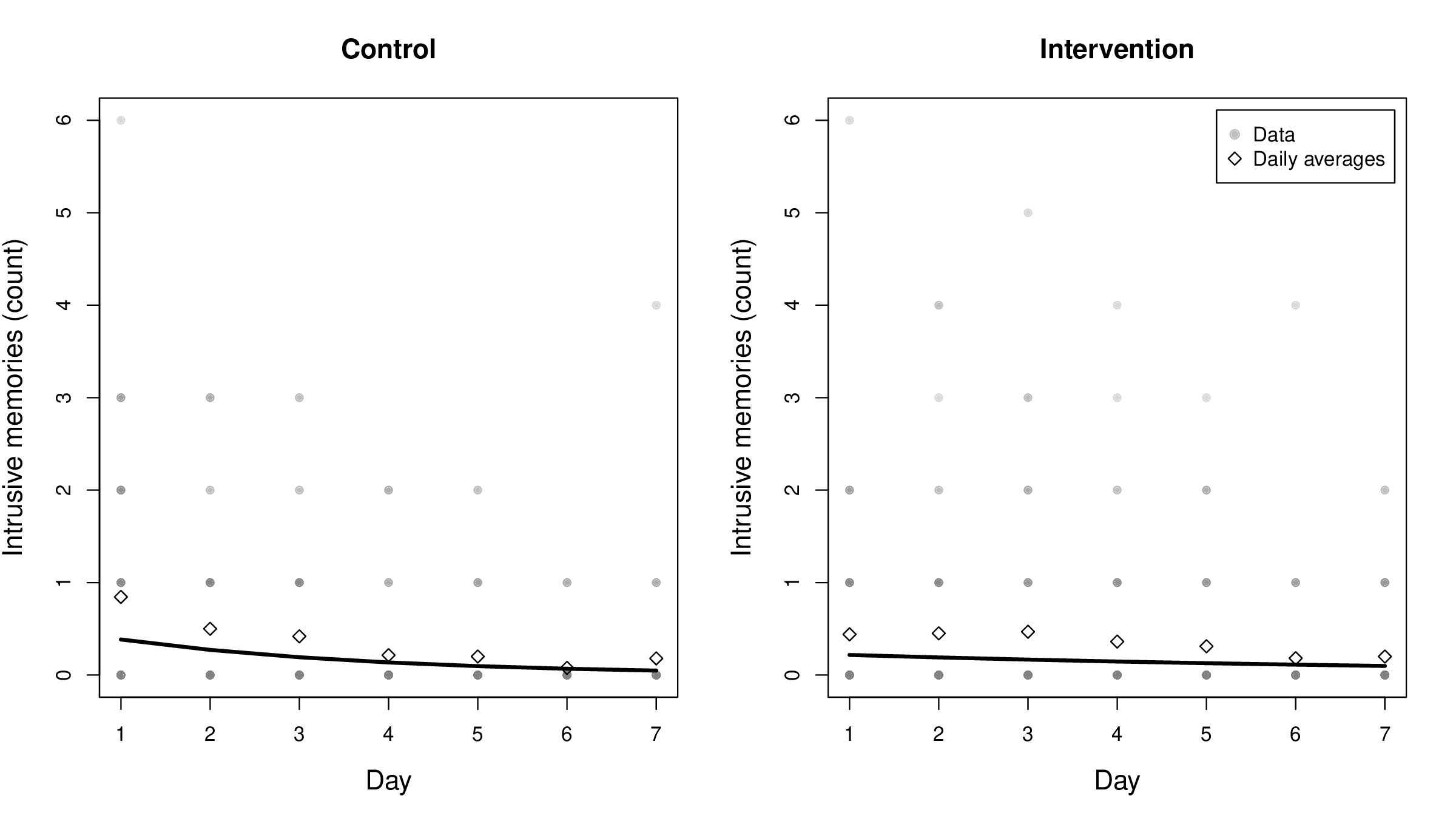


**Supplementary Figure 1. Daily counts of intrusive memories in the control (left panel, n = 45) and intervention (right panel, n = 51) groups. Similar daily counts across subjects are marked darker.**
